# Hypertension management for community-dwelling older people with diabetes in Nanchang, China: study protocol for a cluster randomized controlled trial

**DOI:** 10.1186/s13063-018-2766-5

**Published:** 2018-07-16

**Authors:** Qiang Tu, Lily Dongxia Xiao, Shahid Ullah, Jeffrey Fuller, Huiyun Du

**Affiliations:** 1 College of Nursing and Health Sciences, Flinders University, GPO Box 2100, Adelaide, SA 5001 Australia; 2 South Australian Health and Medical Research Institute, Adelaide, SA Australia

**Keywords:** Hypertension, Diabetes, Cluster randomized controlled trial, China, Collaborative care, Primary health system

## Abstract

**Background:**

Although China has a large number of older people living with diabetes and hypertension, the primary care system is underdeveloped and so management of these conditions in community care settings is suboptimal. Studies have shown that the collaborative care model across care settings that address both pharmacology and nonpharmacology interventions can achieve hypertension control for older people with diabetes. Barriers to implementing and evaluating this model of care are widely recognized in low and middle-income countries including China.

This study will therefore test the hypothesis that a hypertension management program built on collaboration between hospitals and community health service centers in China can improve blood pressure control in people aged 60 years and older with diabetes as compared to usual care.

**Methods:**

A cluster randomized controlled trial will randomly allocate 10 wards from four hospitals in Nanchang to either an intervention group (*N* = 5) or a usual care group (*N* = 5). At least 27 participants will be recruited from each ward and the estimated sample size will be 135 patients in each group. The intervention includes individualized self-care education prior to discharge and 6-month follow-up in community health service centers. Health professionals from both hospitals and community health service centers will be resourced to collaborate on the implementation of the postdischarge interventions that reinforce self-care. The primary outcome is systolic blood pressure at 6-month follow-up adjusted for baseline value. Secondary outcomes are self-care knowledge, treatment adherence, HbA1c and lipid levels, quality of life, the incidence of adverse events and the incidence of unplanned hospital readmission at 6-month follow-up adjusted for baseline value. A multilevel mixed-effect linear regression model will be used to compare the changes in health outcomes between the intervention and usual care groups.

**Discussion:**

This study will determine whether collaborative care among health professionals between hospitals and community health service centers will improve hypertension management for older people with diabetes in the study sites. The program, if effective, will have an immediate application to hypertension management in the healthcare system in China.

**Trial registration:**

Australia New Zealand Clinical Trials Registry, ACTRN12617001352392. Retrospectively registered on 26 September 2017.

**Electronic supplementary material:**

The online version of this article (10.1186/s13063-018-2766-5) contains supplementary material, which is available to authorized users.

## Background

It is estimated that one third of adults (over 100 million) with type 2 diabetes around the world live in China [1]. One third of the Chinese population are expected to be aged 60 years and older (430 million people) by the year 2050 [2]. The continuous growth of the older population will inevitably increase the incidence of hypertension and diabetes in this population group, resulting in an increased burden on the healthcare system. Hypertension frequently coexists with diabetes. It is reported that 50–80% of patients with diabetes are affected by concurrent hypertension [3]. People with comorbidity of diabetes and hypertension are associated with two times higher risk of developing cardiovascular disease and 7.2 times higher mortality rate compared to those who have diabetes only [4, 5]. Clinical studies have indicated that cardiovascular events and hypertension-related mortality in patients with diabetes can be significantly reduced by optimal hypertension management [6, 7]. However, in China only 14.9% of patients with coexisting hypertension and diabetes have their blood pressure controlled and the disease burden on the health systems is widely reported [8]. Although a primary care approach to hypertension management is well recognized as the most cost-effective way to reduce the disease burden [9], there are no practice guidelines for hypertension management at the community level in China and evidence on how to support the development of this care approach is lacking. This study will add research evidence to the international community to inform practice by exploring a postdischarge intervention to improve hypertension management for older people with diabetes codelivered by health professionals working in hospital and primary care settings in Nanchang, China.

Factors associated with uncontrolled hypertension among older people with diabetes include patient factors (i.e., the lack of knowledge, skill and resources to perform self-care) and healthcare system factors (i.e., the lack of continuity of care and collaboration between different care providers) [2, 10, 11]. Poor communication and collaboration between hospitals and community health service centers exists in many low and middle-income countries which leads to fragmented healthcare delivery for discharged patients [12–14]. Although community health service centers have been established in China to strengthen primary care, collaboration between these centers and hospitals is underdeveloped. A study on healthcare referral services in four cities of China, including Nanchang, revealed that 57% of GPs never had communication with specialists [15]. A population-based study showed that only 34% of residents considered the use of community health service centers in Changsha, China [16]. The main factors contributing to the underutilization of primary care appear to be the lack of effective mechanisms to enable referrals to GPs from hospital specialists, and the lack of communication and collaboration between hospital care and primary care providers [12, 15].

In this study context, after hospital discharge, patients who are not managed by GPs and community nurses usually seek treatments in hospitals only when their conditions get worse [13]. As limited time is available for each patient, the treatment at hospitals mainly focuses on medication, but without attention to nonpharmacological interventions, such as health education to improve patients’ self-care capabilities. This acute and episodic treatment is not in line with contemporary self-care principles that encourage patients to actively engage in their chronic condition management through goal-setting and regular review [17]. Improvements in referral to and capacity-building of the primary care system will enable patients to use primary care as the main source of care to improve both pharmacological and nonpharmacological interventions.

A body of evidence shows that up to 50% of community-dwelling older people in the USA had one or more geriatric conditions such as incontinence, falls, malnutrition, cognitive impairment and pressure ulcers [18, 19]. These conditions compound the complexity of hypertension and diabetes, and contribute to adverse outcomes and hospital admission [20]. Community-dwelling older people who are susceptible to or have these geriatric conditions require primary care services to achieve a long-term control of these conditions, rather than relying on irregular outpatient clinic visits or admission to hospital.

Studies have demonstrated that close collaboration between hospitals and community health service centers can provide patients with continuous and integrated care delivery, which is recognized as an effective approach to improve quality of care, decrease healthcare utilization and reduce costs associated with hypertension treatment [12, 21, 22]. Randomized controlled trials in high-income countries with well-developed healthcare systems and health insurance schemes have demonstrated that multifactorial interventions delivered by a multidisciplinary team involving nurses and doctors had positive effects in hypertension management for older people with diabetes [23–25]. This management often incorporates the following components: health education at hospital and community healthcare settings, healthy lifestyles (i.e., weight loss, smoking cessation, salt and alcohol restriction, increased physical activity), medication adherence and intensification, timely medication adjustment and complication prevention, and continuous and rigorous follow-up visits in primary care [23, 26–29]. Reports of intervention programs including a combination of these components remain scarce in the low and middle-income countries with lower health resources in primary care such as China.

In this study, collaboration between hospitals and community health service centers will be developed to promote a primary care approach to managing hypertension for older people with type 2 diabetes. This collaboration will support the capacity-building of the primary care system in chronic disease management. We hypothesize that a hypertension management program built on collaborative care among health professionals within and between hospitals and community health service centers will improve blood pressure control among community-dwelling older people with diabetes in Nanchang, China.

## Methods

### Study design

**Fig. 1:**
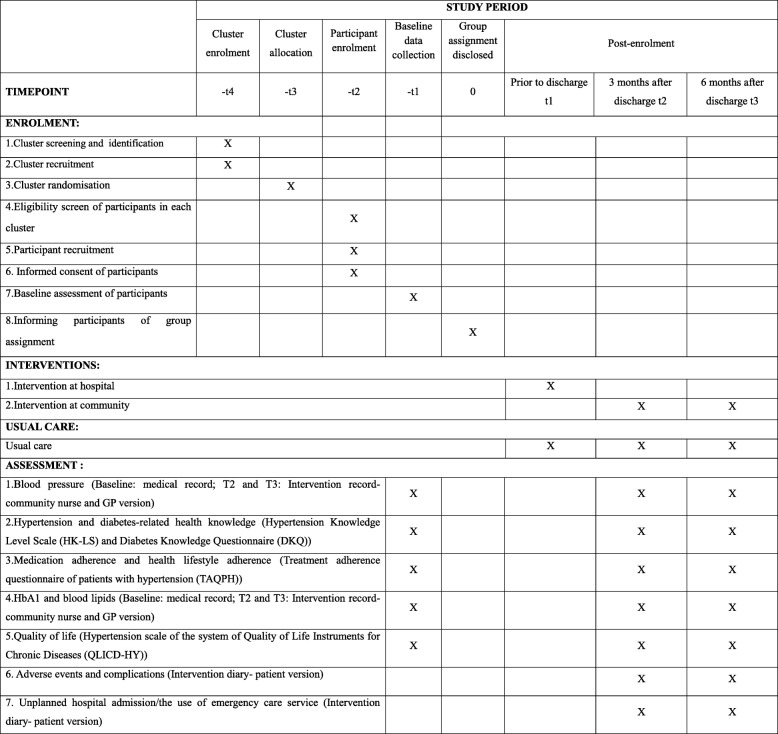
SPIRIT figure. GP general practitioner, HbA1 glycosylated hemoglobin

**Fig. 2:**
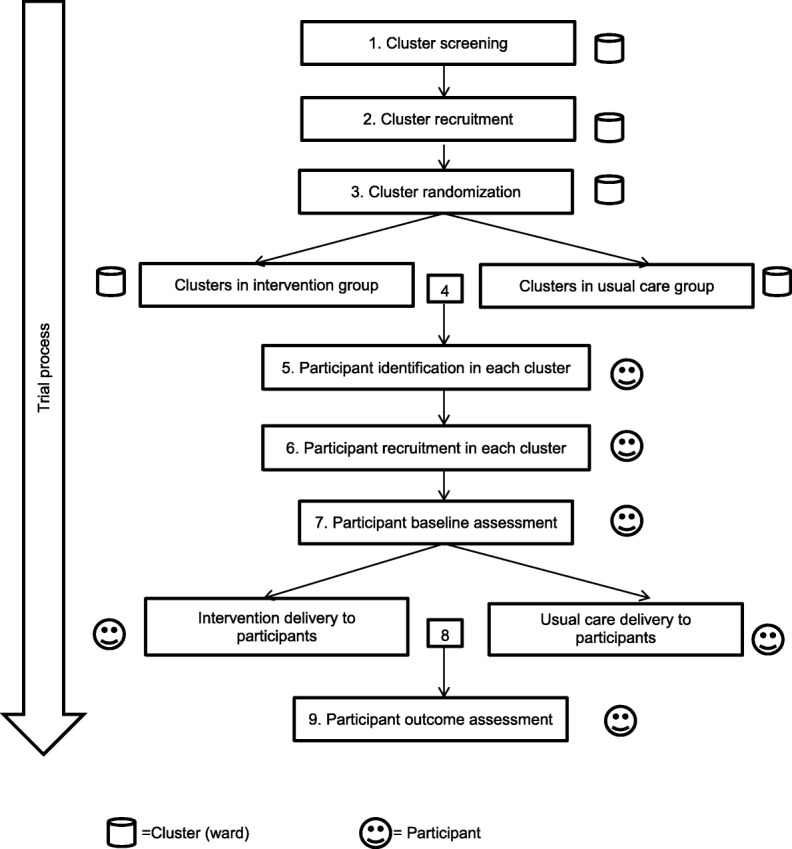
Timeline diagram

A cluster randomized controlled trial will commence at hospitals via discharge and then continue at community health service centers for 6 months in Nanchang, the capital city of Jiangxi Province in China. This cluster design will minimize the risk of contamination between intervention and usual care groups and reflect the classification of public hospitals in China at three levels, namely community health service centers, secondary hospitals and tertiary hospitals [30]. Tertiary hospitals are the final referral hospitals and provide more comprehensive care services for patients compared with secondary hospitals. The Standard Protocol Items: Recommendations for Interventional Trials (SPIRIT) that provide an overview of the schedule of enrolment, interventions and assessments are presented in Fig. 1. The SPIRIT checklist is shown in Additional file 1. The timeline diagram is presented in Fig. 2.

### Ethical approval and consent to participate

The study protocol was approved by the Southern Adelaide Clinical Human Research Ethics Committee in Australia (Approval Number: 345.16) and the Department of Scientific Research Management at the Health and Family Planning Commission of Jiangxi Province, China. All participants and health professionals delivering the interventions will be given written information so that they can provide informed consent. Each participant and participating health facility will be given a unique numerical code to ensure anonymity. Information provided by participants will be collected in a de-identifiable form and treated confidentially. Data collected through this study will be stored in a secure area in the University that the first author is enrolled in a PhD program. All study-related data will only be accessible to the researchers.

### Eligibility: inclusion criteria and exclusion criteria

Hospitals are eligible for recruitment if they have cardiovascular wards and geriatric wards that predominantly provide care services for older people with hypertension. Participants from these wards are eligible for recruitment if: (1) they receive care for both type 2 diabetes and hypertension from one of the four hospitals selected for the study; (2) the reason for hospitalization is uncontrolled hypertension and/or associated complications; (3) they are diagnosed with coexisting type 2 diabetes and hypertension; (4) they are aged ≥ 60 years; (5) they are fit for discharge justified by the specialist; (6) they are without cognitive impairment (assessed by the Mini-Mental State Examination); and (7) they reside in residential areas where the six community health service centers provide care services.

Participants will be excluded if they are: (1) diagnosed with type 1 diabetes; (2) unable to participate in the study because of severe organ damage, disability, cognitive impairment and other life-threatening disease; (3) unwilling to return to a community health service center for follow-up visits; and (4) living outside of these six communities.

### Randomization

**Fig. 3:**
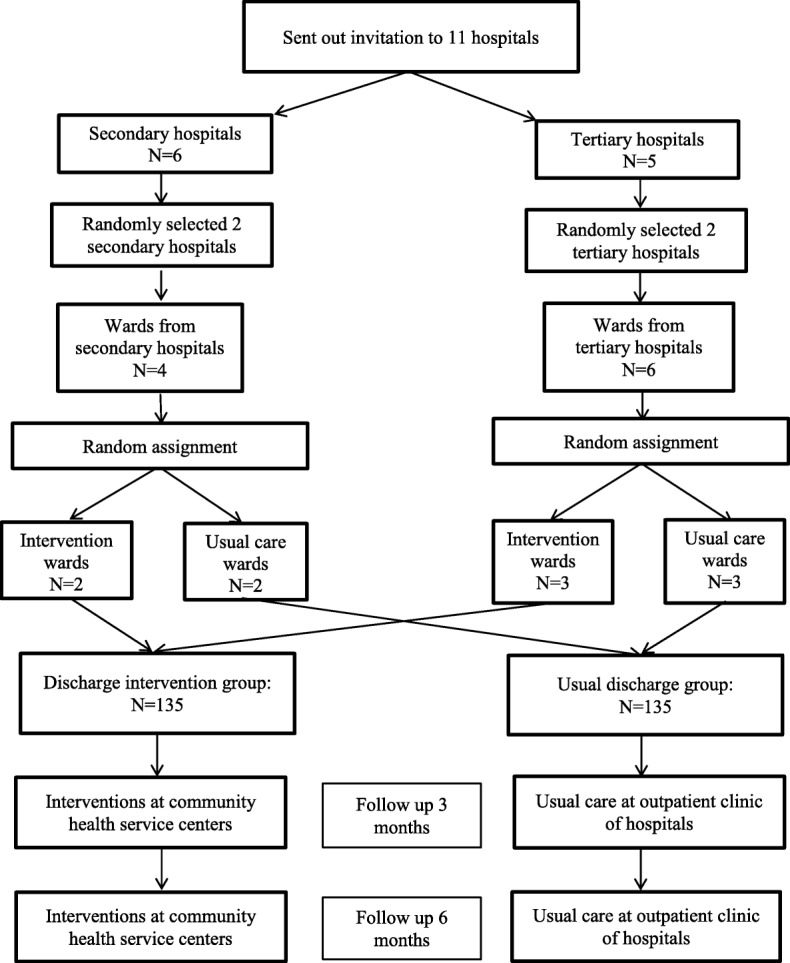
Flow chart of the randomization

Participants will be recruited from hospitals and continue the trial at community health service centers. Randomization will be performed based on the ward clusters rather than participants. Health professionals involved in the study practice are in a single ward only, therefore the chance of contamination between intervention and usual care wards in the same hospital is very low. There are six secondary hospitals and five tertiary hospitals that provide in-patient care for patients with diabetes and hypertension in Nanchang. As part of the ethics application processes, researchers in the project sent out invitations to all of these 11 public hospitals in Nanchang and informed them that two secondary hospitals and two tertiary hospitals would be randomly selected. The number of hospitals to be included in the trial was estimated on the sample size calculation. In total, six secondary hospitals and five tertiary hospitals agreed to participate. To ensure the selected hospitals are representative of the hospitals in Nanchang, two hospitals will be randomly selected from each level. There are two eligible wards in each participating secondary hospitals and three eligible wards in each tertiary hospital. To ensure the selected wards are representative of the wards in each level of the selected hospitals, three wards from tertiary hospitals and two wards from secondary hospitals will be randomly assigned to either an intervention group or a usual care group. The group assignment will be undertaken by a PhD fellow who is not involved in the trial and is blind to the wards. Patients who meet the inclusion criteria in these participating wards will be invited to the trial. There are six community health service centers that provide chronic disease management for residents in Nanchang. The invitations were sent to these community centers and all of them agreed to participate in the trial. A flow chart of the randomization is shown in Fig. 3.

### Description of the interventions

**Fig. 4:**
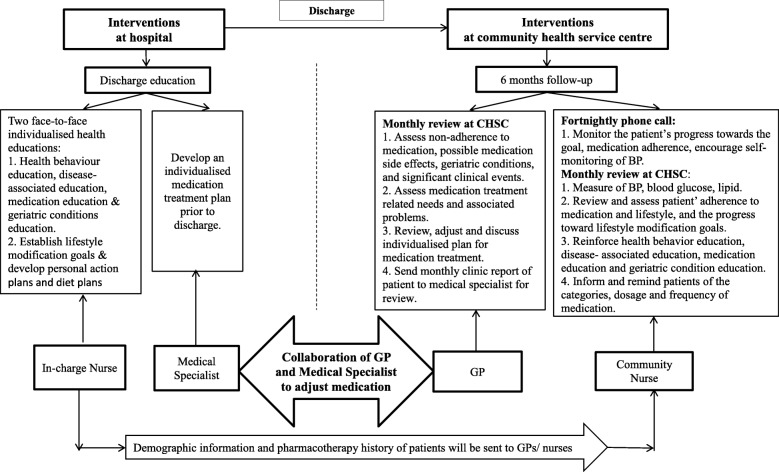
Flow chart of the intervention protocol. BP blood pressure, CHSC community health service center, GP general practitioner

The interventions were developed from a comprehensive literature review about effective hypertension management for older people with diabetes. The intervention will start from the hospital with individualized discharge education provided by the patient’s in-charge nurse and the medical specialist (first stage of intervention). After patients are discharged, and in addition to the usual follow-up in the hospital outpatient clinic, they will be referred to the community health service centers to receive the follow-up intervention over 6 months provided by general practitioners (GPs) and community nurses (second stage of intervention). Health professionals from both hospital and community health service centers will be resourced to work in a collaborative way to implement the postdischarge interventions. A flow chart of the intervention protocol is shown in Fig. 4 and discussed in the following.

#### Stage 1: interventions at hospital

1.1. Individualized discharge education.
  Patients will be given two face-to-face individualized health education sessions specific to diabetic patients with hypertension by the in-charge nurse. The health education includes health behavior education, disease-associated education and medication education with 15 min for each session. A geriatrics condition screening will also be undertaken using the widely recognized evidence-based screening tool “Fulmer SPICES Comprehensive Assessment Tool for Older Adults” [31]. Education on preventing and managing geriatric conditions will be provided by the in-charge nurse based on the findings from the screening.
1.2. Lifestyle modification goals: intervention diary—patient version.
  1.2.1. Patients will be asked to establish lifestyle modification goals and develop personal action plans with the in-charge nurse. Patients will be provided an “intervention diary—patient version” (Additional file 2) to document their lifestyle modification goals, personal action plans, progress toward the goals, medication adherence, adverse events and unplanned hospital admissions/use of emergency care services.
  1.2.2. Patients will be asked to discuss and establish their diet plan with the in-charge nurse. The diet plan will be recorded in the “intervention diary—patient version”.
1.3. Individualized medication treatment.
  Prior to discharge, patients will be given an individualized medication treatment plan by the medical specialist. Individualized medication treatment will be recorded in the “intervention diary—patient version”.
1.4. Referral to the community health service center: intervention record—community nurse and GP version.
  1.4.1. When patients are discharged from hospital, they will be referred to a community health service center by the in-charge nurse for regular follow-up over 6 months. An “Intervention record—community nurse and GP version” (Additional file 3) will be sent to the community nurse by the in-charge nurse on discharge to record the required postdischarge interventions and outcomes.
  1.4.2. In order to establish collaboration and maintain communication among the doctor and nurse at the hospital, the patient and the GP and nurse at the community health service center, a two-way referral letter has been developed (Additional file 4).
  1.4.3. The in-charge nurse at the hospital will contact the GP and nurse in the community health service center through a telephone call and will fill in the two-way referral letter for patients.
  1.4.4. The in-charge nurse will help patients to contact the nearby community health service center for the follow-up visit. Patients will be advised of the community health service center they have been referred to, and the name and contacts of the GP and nurse in the community health service center.
  1.4.5. Data in relation to the patient’s demographic information, pharmacotherapy history and other clinical information will be sent to the community nurse by the in-charge nurse on discharge.
  1.4.6. Patients will take the referral letter and discharge abstract to the community health service center for the follow-up visit.
1.5. Monitoring.
  The in-charge nurse at the hospital will keep track of the patient’s referral progress through making telephone calls with the patients and community nurses to ensure patients are seen at the community health service center.

#### Stage 2: regular follow-up interventions over 6 months at community health service centers

2.1. Fortnightly telephone call.
  Patients will receive a fortnightly telephone call at home from the community nurse to monitor their progress toward setting goals and medication adherence.
2.2. Monthly community health service center visit.
  Patients will be asked to visit the community nurse and GP at the community health service center every month for follow-up visits.
  2.2.1. Patients will receive blood pressure measurement every month by the community nurse.
  2.2.2. Patients will receive a review and assessment from the community nurse on their adherence to medication and lifestyle recommendations, and their progress toward lifestyle modification goals.
  2.2.3. Patients will receive reinforced health education from the community nurse to improve their self-management ability and treatment adherence. Patient will be given health education on preventing or treating geriatric conditions based on the “Fulmer SPICES Comprehensive Assessment Tool for Older Adults”.
  2.2.4. Patients will receive an assessment from the GP on their clinical outcomes, problems of nonadherence to medication, medication side effects, drug therapy problems, drug-related needs and clinical events occurred.
  2.2.5. Patients will be asked to discuss their individualized treatment plan and medication adjustment with the GP.
  2.2.6. Patients will be asked to document their medication changes in the “intervention diary—patient version”.
  2.2.7. Patients will be reminded of the categories, dosage and frequency of medication required to be taken prior to the next visit by community nurse.
2.3. GP and medical specialist collaboration.
  The GP will discuss with the medical specialist at the hospital via email or telephone when necessary to adjust medication. BP, HbA1c and lipid readings will be recorded by the community nurses and formatted as a monthly report and then sent to medical specialists for review. Urgent symptoms will be communicated to the medical specialist immediately for additional orders by the GP.

### Usual care

In the usual care group, patients will receive routine discharge education at hospital. After they are discharged, they will go to their usual outpatient clinic of the hospital to receive follow-up and outcome measures at 3 and 6 months. They will not be referred to the community health service center. They will be asked to record the number of adverse events and unplanned hospital admissions/use of emergency care services during 3-month and 6-month follow-up in a usual care table (Additional file 5). The researcher will record the HbA1c and lipid levels close to the 3-month and 6-month follow-up from the patients’ medical record.

### Outcome measures

#### Primary outcome

The primary outcome is systolic blood pressure at 6-month follow-up adjusted for baseline value. At each measurement point, blood pressure will be measured three times at 5-min intervals on the participant’s right arm in a sitting position. The average of the three blood pressure readings will be calculated as the final BP reading for each group at each time point, which minimizes measurement error.

#### Secondary outcomes

Secondary outcomes are the following outcomes at 6-month follow-up adjusted for baseline value: (1) hypertension and diabetes-related health knowledge score measured by a combination of the Hypertension Knowledge-Level Scale (HK-LS) [32] and the Diabetes Knowledge Questionnaire (DKQ) [33]; (2) medication adherence and health lifestyle adherence score measured by the Treatment Adherence Questionnaire of Patients with Hypertension (TAQPH) [34]; (3) HbA1c and lipid values; (4) quality of life score measured by the hypertension scale of the Quality of Life Instruments for Chronic Diseases (QLICD-HY) [35]; (5) the incidence of adverse events and complications associated with hypertension, diabetes, treatments and geriatric syndromes recorded by patients/or carers in the “intervention diary—patient version”; and (6) the incidence of unplanned hospital admissions/use of emergency care services due to uncontrolled hypertension, diabetes and geriatric syndromes recorded by patients or carers in the “intervention diary—patient version”.

Through the study, we hope to identify the challenges that patients face in their hypertension management in the community healthcare settings and to evaluate how to overcome these challenges by improving the capacity of the primary care system. Hence, two open-ended questions will be included for patients upon study completion: (1) Are you satisfied with the follow-up provided by the GP and community nurse in the community health service center? (If not, please explain the reason); and (2) Did you encounter any difficulties of care in the community health service center after discharge? (If yes, please list the difficulties you encountered).

### Sample size

The sample size calculation is based on the primary outcome and is estimated on the basis of an earlier randomized controlled trial study in relation to hypertension management for diabetic patients [36]. In this earlier study, the researchers found a 7.3 mmHg significant reduction in systolic blood pressure (SBP) with a standard deviation of 12.1 mmHg and an intraclass correlation coefficient (ICC) of 0.05 in the intervention group [36]. Since randomization in our study will be done by wards (clusters), the sample size has been adjusted to take into account the design effect. Our study is designed to have 80% power to detect a difference of 7.3 mmHg SBP between the group means when the standard deviation is 12.1 mmHg. This assumes that a sample size of five clusters per group with 25 participants per cluster will complete the study and the ICC is 0.05, giving a design effect of 1.48. The ICC also reflects the situation that the hospitals in the study are from the same city under the same hospital administration standard; therefore, each cluster is unlikely to differ from the others with respect to the variable of interest. Each cluster will be of equal size. Assuming an attrition rate of 10%, we would require 27 participants per cluster. In total, 10 clusters with 270 participants will be recruited.

### Participant recruitment

Recruitment posters will be presented in the patient activity rooms in the participating wards. An information pack including an information sheet, consent form and other study-related documents (i.e., intervention diary—patient version; usual care table) will be displayed on a table placed under the poster for potential participants to view. Potential participants will be asked to return their decision on participation via a response slip to a drop box in each participating ward. On receiving the response slip, a research recruiter who is blinded to the group allocation and has no interest in the outcome of the study will contact the patients to confirm their eligibility to participate. One of the project team members, who will know the allocation, will provide detailed information related to the study and have the participants sign the consent form. Although the allocation will be known to the project team member, the risk of selection bias and influences on the measure of baseline outcome will be minimal because the participants will have been identified and recruited by the research recruiter rather than the project team member. Participants will be informed that refusal to participate in this study will not alter the caring relationship between them and the hospitals. Participants will be reminded that they can discontinue participation in the study at any time without any influences on their usual care.

### Data collection

At baseline, the in-charge nurse will distribute the questionnaires to participants or their family caregiver. They will complete the questionnaires once the consent form is signed and before the individualized discharge education is provided. The patient’s in-charge nurses at the hospital will provide assistance to participants should they have any questions about the questionnaire. Clinical outcomes (BP, HbA1c and lipid values) will be directly recorded from the health record of patients by a research assistant.

At 3-month and 6-month follow-up, community nurses will distribute the questionnaires to participants in the intervention group when they visit the community health service center. Community nurses will be available to assist patients should they have any questions about the questionnaire. In the usual care group, a nurse in the medical specialist clinics will distribute the questionnaire to participants when they visit the outpatient clinic at 3-month and 6-month follow-up. The nurse will be available to assist patients should they have any questions.

Completed questionnaires will be placed in a prepared sealable envelope to maintain confidentiality. The researcher will prepare a drop box in each ward, community health service center and outpatient clinic. Participants will return the sealed questionnaires to the drop box. The research assistants will collect completed questionnaires on a weekly basis.

### Quality control procedures

Quality of data collection will be ensured during the whole study phases. Two 1-h workshops and ongoing support at each participating site will be provided to the health professionals to allow them to discuss, understand and follow the study protocol. Research assistants will be employed to distribute and collect the questionnaires. Consent forms, questionnaires and other data collection documents will be checked for completeness. Data at three time points (baseline, 3-month and 6-month follow-up) will be checked using the birth date of patients to ensure the data are matched correctly. The quality of the study will be overseen by the PhD supervision team and the representative of the Department of Scientific Research Management at Health and Family Planning Commission of Jiangxi Province, China. The data entered into SPSS will be checked to prevent input errors by the other two PhD fellows who are not involved in this study. The compliance of patients with the interventions will be optimized by a telephone call from the nurse at the community service center to remind them of the follow-up visits at the community health service center.

### Blinding and allocation concealment

Participants will not know their group allocation during the recruitment and baseline data collection. The research recruiter who is blinded to the group allocation and has no interest in the study will assist the recruitment of participants. Allocation will be disclosed to participants after the baseline data collection. Due to the nature of the study interventions, health professionals who deliver the intervention will know the group allocation during the trial. However, the statistician involved in the data analysis is blinded to the group allocation.

### Intervention fidelity

Intervention fidelity refers to the extent that proposed interventions are delivered as planned in the protocol. The items of interventions recorded by both patients and community health professionals include: (1) intervention diary—patient version; and (2) intervention record—community nurse and GP version. Compliance with required interventions will be matched using these two tools.

### Dealing with contingencies

Possible contingencies are the following: (1) patients refuse to participate in the project; the strategy to deal with this will be to extend the recruitment period to gain sufficient sample size; and (2) unexpected tasks that health services need to deal with, such as outbreak of infectious disease; the strategy to deal with this will be to temporarily cease the trial and recommence when the health service returns to normal. The Department of Scientific Research Management at Health and Family Planning Commission of Jiangxi Province, China, will deal with the contingencies that may occur during the study.

### Statistical methods

Statistical analysis will be performed using Statistical Package for Social Science (SPSS) version 23 and STATA software version 14. To test the effectiveness of the intervention on the primary and secondary outcomes, a multivariate multilevel mixed-effects linear regression model will be used to analyze outcomes. This is due to the hierarchical structure of the data and is based on the consideration of the correlations between participants’ repeated measurements over time. Because the participants are nested within hospital wards, we will fit a three-level mixed model with random intercepts at both the cluster and the participant-within-cluster levels. Thus, models will account for the clustering in wards, participants within clusters and over time using the *mixed* command. Wards and participants within wards will be treated as random effects. The main effects will be group (intervention or comparison), time (baseline, 3 and 6 months) and group × time interaction. A small-sample correction to the restricted maximum likelihood estimator will be used to improve the inference for the fixed parameters and is available in STATA *mixed* command. Models will be adjusted by baseline value and confounders that include age, gender, body mass index (BMI), duration of hypertension, duration of diabetes, smoking status and education level [37, 38]. A univariate model will also be used for unadjusted estimates. The level of significance will be set at *p* < 0.05. Where appropriate, 95% confidence intervals (95% CIs) will be reported along with *p* values.

Data analysis will be performed according to the intention-to-treat principle. Participants who withdraw or do not complete the 6-month follow-up will be included in the analysis. A multiple imputation technique will be used for large missing data.

## Discussion

The transition between hospital care and community care is a critical period in the care of elderly patients with diabetes and hypertension who often have complex medical problems. The incidence rate of adverse events after discharge and the readmission rate are high due to the inappropriate self-care behaviors of patients and lack of timely monitoring of patients’ conditions by health professionals [11, 39]. It has been reported that within a week after hospital discharge, primary health care is required by 80% of patients [11]. Continuity of care through follow-up in primary care as the first contact for patients after discharge can bring positive outcomes for older patients [11, 26]. The transition from passive care to proactive and preventive care can be achieved through establishing a care continuum in the current health systems in China and other low and middle-income countries. Currently, most patients in China only visit doctors when the symptoms of diseases appear. Many complications do not show symptoms in the early stages when they are easier to treat and so the best treatment period may be delayed or missed. By establishing continuous care after discharge, disease-related complications can be prevented or detected earlier and so promptly treated in the primary care system.

As recommended by the World Health Organization [14], patients with chronic conditions require long-term, comprehensive and coordinated care after discharge that is easy to access and provided by the primary health system. A well-developed primary care system contributes to alleviating disparities in healthcare utilization [40]. In 2015, the Chinese government issued an “Outline for the Planning of the National Medical and Health Service System (2015–2020)” which emphasizes collaboration between hospitals and community health service centers. The purpose is to improve prevention and management of chronic disease in primary care settings, reduce hospital care burden and lower national medical costs. This study is in line with these national health reforms that transfer the focus of care from hospitals to community health settings. Interventions introduced in this trial will build capacity of the primary care system to provide accessible and quality care for elderly people with chronic conditions. Interventions in the trial will enable older people to easily access health care a short distance from their home instead of waiting in long queues at a hospital. Collaboration between hospitals and community health service centers can bridge the gap between service capacity of community health professionals and residents’ demand for quality in healthcare.

Hypertension and diabetes management after discharge is a process that requires lifelong adjustments to nonpharmacological and pharmacological interventions due to the chronic nature of these conditions. Lack of adherence to pharmacological and nonpharmacological treatment has been identified as a key barrier to optimal hypertension control. Medication adherence is positively associated with hypertension control and reduction in the risk of complications [41]. However, only 20–60% patients in China comply with antihypertensive drugs [42]. Nearly half of patients with diabetes and hypertension are likely to discontinue their medication treatment in the first 6 months after discharge or when their symptoms diminish [2, 42]. Among the patients who adhere to medications, 50% of them take their medications in an incorrect way [2]. Therefore, long-term medication management is required.

As a chronic disease, hypertension requires life-long medication treatment. Older patients whose memory function decline are more likely to forget taking medications as prescribed and to experience side effects and adverse reactions. Inappropriate medication management can lead to repeat hospital admission. In a study involving 86 patients who were hospitalized, 49% of hospital admissions were due to medical error [11]. Therefore, it is important to prevent medication-related problems. In this study, health professionals at community health service centers will regularly monitor patients’ medication compliance, deal with the side effects of medication and adjust medical therapies to achieve continuity in optimal medication management.

Therapeutic lifestyle changes (TLC) have demonstrated effectiveness in the control of hypertension and improved quality of life among older people [28]. However, a community-based cross-sectional study in Alexandria found that only one in 10 patients can follow the recommended lifestyle modifications such as dietary change, exercise adjustment and weight control [43]. Factors associated with poor self-management include a lack of understanding of the care plan and low confidence and motivation to make healthy choices. Health education can address these barriers by equipping patients with the motivation, self-care skills and knowledge to manage their chronic conditions. Considering the low level of health knowledge among older people, a sustainable and structured follow-up, including regular health education (involving positive behavior changes and medication compliance), and professional health counseling are required for older patients who are discharged. In addition, the monthly monitoring of conditions for older people in community settings can ensure early detection of their functional decline, which prevents hospital readmission. A study in Canada found that hypertension-related hospital admissions of older patients was reduced by 9% during 10 weeks by implementing a community-based education program [42]. Therefore, a sustainable health system is built through promoting service improvements in primary care, such as intensive follow-up to support older patients to maintain their adherence to medical treatment and healthy lifestyle.

Previous hypertension management programs in China have been mostly hospital based or community based and only target either pharmacology or nonpharmacology interventions [8, 26]. This is first trial in China to build integrated care based on collaboration and coordination between hospitals and community health service centers to support older people to manage diabetes and hypertension at home using multifactorial interventions. In addition, this study synthesizes the latest evidence and includes the combination of effective intervention components on hypertension management for people with diabetes, which differs from the previous studies in China that only tested single intervention components [44, 45].

The program, if effective, will have an immediate application to hypertension management in the healthcare system in China. In addition, this study uses hypertension and diabetes as an entry point to improve chronic disease management in primary care. Success of this intervention program, that transfers patients from hospital to community health service centers, can also be applied to the management of other chronic diseases for hospitalized patients.

Limitations of this study are the following. First, the trial will be undertaken in 10 clusters only and hence sampling bias may occur, which means that the findings may not represent the population. Second, as an open-label study, the patients and health professionals will be aware of the groups they are assigned to and their allocated treatment during the trial. Patient bias may occur when they complete the self-reported questionnaires. Third, the duration of follow-up at the community health service center is relatively short (only 6 months). The long-term effects of this intervention program will need to be explored in a future study.

## Trial status

At the time of submission of the manuscript, this trial is in progress and participants are being recruited. The study began in June 2017, and is expected to be complete by June 2018.

## Additional files

Additional file 1: SPIRIT checklist. (PDF 806 kb)

Additional file 2: Intervention diary—patient version. (DOCX 27 kb)

Additional file 3: Intervention record—community nurse and GP version. (DOCX 30 kb)

Additional file 4: Two-way referral letter. (DOCX 21 kb)

Additional file 5: Usual care table. (DOCX 19 kb)

## Abbreviations

GP: General practitioner
HbA1C: Glycosylated hemoglobin
