# Supplementary material for: Hypertension management for community-dwelling older people with diabetes in Nanchang, China: study protocol for a cluster randomized controlled trial
Source: Trials. 2018 Jul 16;19:385. doi: 10.1186/s13063-018-2766-5 (PMC6048858; doi:10.1186/s13063-018-2766-5)
Supplement: Supplementary file 2 — Intervention diary—patient version. (DOCX 27 kb) [file 13063_2018_2766_MOESM2_ESM.docx]

**Additional file 2: Intervention Diary- patient version** (Please tick ✓in the spaces of the items you have done prior to each visit)

Code of patient: ________ Code of health facility: ________

| **Discharge planning** | | | | | **Self-management record after discharge** | | | | | |
| --- | --- | --- | --- | --- | --- | --- | --- | --- | --- | --- |
| **Contents** | | | | | **1^st^ month**  **__D__M__Y** | **2^nd^ month**  **__D__M__Y** | **3^rd^ month**  **__D__M__Y** | **4^th^ month**  **__D__M__Y** | **5^th^ month**  **__D__M__Y** | **6^th^ month**  **__D__M__Y** |
| Health behaviour goal and plan made on the discharge | Diet | |  | | Completed ☐    If uncompleted,  __times of non-adherence to goals |  |  |  |  |  |
|  | Alcohol | |  | | Completed ☐    If uncompleted,  __times of non-adherence to goals |  |  |  |  |  |
|  | Physical exercise | |  | | Completed ☐    If uncompleted,  __times of non-adherence to goals |  |  |  |  |  |
|  | Smoking | |  | | Completed ☐    If uncompleted,  __times of non-adherence to goals |  |  |  |  |  |
|  | Positive emotions | |  | | Completed ☐    If uncompleted,  __times of non- adherence to goals |  |  |  |  |  |
|  | Geriatric conditions | |  | | Completed ☐    If uncompleted,  __times of non- adherence to goals |  |  |  |  |  |
|  | Other | |  | | Completed ☐    If uncompleted,  __times of non-adherence to goals |  |  |  |  |  |
| BP reading of self-monitoring | | | | | 1^st^ week_______  2^nd^ week_______  3^rd^ week_______  4^th^ week_______ |  |  |  |  |  |
| Medication use and its adherence | | 1.Name of medication | |  | no missed ☐ |  |  |  |  |  |
|  |  | Dosage and Frequency | | _____times/day  _____tablet/each time | _______times of missed medication this month  Reasons of medication non adherence___________________  ____________________________ |  |  |  |  |  |
|  |  | 2.Name of medication | |  |  |  |  |  |  |  |
|  |  | Dosage and Frequency | | _____times/day  _____tablet/each time |  |  |  |  |  |  |
|  |  | 3.Name of medication | |  |  |  |  |  |  |  |
|  |  | Dosage and Frequency | | _____times/day  _____tablet/each time |  |  |  |  |  |  |
| Adverse events | | falls and fall-related injuries ☐  pins and needles ☐  dizziness ☐  headache ☐  sleep difficulties ☐  swelling of legs or ankles ☐  chest distress ☐  stomach disorder ☐  backache ☐  cough ☐  dyspnoea ☐  vomit ☐  others (please specify) ______ | | |  |  |  |  |  |  |
| Numbers of unplanned hospital admission/ the use of emergency service after discharge | | | | |  |  |  |  |  |  |
| Date for the next visit | | | | | ___D___M___Y |  |  |  |  |  |
