# Supplementary material for: Hypertension management for community-dwelling older people with diabetes in Nanchang, China: study protocol for a cluster randomized controlled trial
Source: Trials. 2018 Jul 16;19:385. doi: 10.1186/s13063-018-2766-5 (PMC6048858; doi:10.1186/s13063-018-2766-5)
Supplement: Supplementary file 3 — Intervention record—community nurse and GP version. (DOCX 30 kb) [file 13063_2018_2766_MOESM3_ESM.docx]

**Additional file 3: Intervention record- community nurse and GP version** (please tick ✓ in the spaces of the items you have done at each visit)

Code of community nurse: __________ Code of GP: ____________ Code of community health service centre: _________

| **Community nurse** | | | | | | | | | | | **GP** | | | | |
| --- | --- | --- | --- | --- | --- | --- | --- | --- | --- | --- | --- | --- | --- | --- | --- |
| **Date** | **Fortnightly phone call** | | **Monthly clinic visit** | | | | | | | | | | | | |
|  | **Length of time** | Monitor the patient’s progress towards the goal, medication adherence, encourage BP self-monitoring | **Length of time** | Measure BP | Health education and establish lifestyle goal | Test health knowledge | Adherence to lifestyle recommendations | Medication adherence | Adverse events and unplanned admission | **Nurse Signature** | Review and discuss individual treatment plan | Medication change (describe) | Discussions with specialists (describe the outcomes) | Refer patient to specialist and its reason | **GP Signature** |
| **1^st^ month**  ____d/m/y | 1^st^ Phone call ____ | Yes  No |  |  |  |  | Complied  always ☐  Complied  often ☐  Complied  sometimes☐  Complied rarely ☐  Non-complied ☐ | Complied  always ☐  Complied  often ☐  Complied  sometimes☐  Complied rarely ☐  Non-complied ☐ |  |  | Issues identified: __________________ |  |  |  |  |
|  | 2^nd^ phone call_____ | Yes  No |  |  |  |  |  |  |  |  |  |  |  |  |  |
| **2^nd^ month**  ____d/m/y | 3^rd^ phone call_____ | Yes  No |  |  |  |  |  |  |  |  |  |  |  |  |  |
|  | 4^th^ phone call_____ | Yes  No |  |  |  |  |  |  |  |  |  |  |  |  |  |
| **3^rd^ month**  ____d/m/y | 5^th^ phone call_____ | Yes  No |  | BP reading _______ |  | Score: ______ | Score: ______ | Score: ______ | Number:­­­­_____ |  |  |  |  |  |  |
|  | 6^th^ phone call_____ | Yes  No |  |  |  |  |  |  |  |  |  |  |  |  |  |
| **4^th^ month**  ____d/m/y | 7^th^ phone call_____ | Yes  No |  |  |  |  |  |  |  |  |  |  |  |  |  |
|  | 8^th^ phone call_____ | Yes  No |  |  |  |  |  |  |  |  |  |  |  |  |  |
| **5^th^ month**  ____d/m/y | 9^th^ phone call_____ | Yes  No |  |  |  |  |  |  |  |  |  |  |  |  |  |
|  | 10^th^ phone call_____ | Yes  No |  |  |  |  |  |  |  |  |  |  |  |  |  |
| **6^th^ month**  ____d/m/y | 11^th^ phone call_____ | Yes  No |  | BP reading _______ |  | Score:  ____ | Score:  ____ | Score:  ____ | Number­­­_____ |  |  |  |  |  |  |
|  | 12^th^ phone call_____ | Yes  No |  |  |  |  |  |  |  |  |  |  |  |  |  |
