# Supplementary material for: Hypertension management for community-dwelling older people with diabetes in Nanchang, China: study protocol for a cluster randomized controlled trial
Source: Trials. 2018 Jul 16;19:385. doi: 10.1186/s13063-018-2766-5 (PMC6048858; doi:10.1186/s13063-018-2766-5)
Supplement: Supplementary file 4 — Two-way referral letter. (DOCX 21 kb) [file 13063_2018_2766_MOESM4_ESM.docx]

**Additional file 4: Two-way referral letter between hospital and community health service centre**

| Participant code： | | Gender： | Birth date： | | Admission number at hospital： |
| --- | --- | --- | --- | --- | --- |
| Telephone number： | | | Home address： | | |
| Discharge | Ward at hospital：_______________________ | | Admission | Community health service centre:_______________ | |
|  | Date of discharge from hospital：  ____day ___month ___ _year | |  | Date of admission to community health service centre:  ____day ___month ___ _year | |
|  | Name of in-charge doctor：________________  Telephone number：_____________________  Name of in-charge nurse：________________  Telephone number：_____________________ | |  | Name of GP：______________________________  Telephone number：_________________________  Name of nurse：____________________________  Telephone number：_________________________ | |
