# Supplementary material for: Hypertension management for community-dwelling older people with diabetes in Nanchang, China: study protocol for a cluster randomized controlled trial
Source: Trials. 2018 Jul 16;19:385. doi: 10.1186/s13063-018-2766-5 (PMC6048858; doi:10.1186/s13063-018-2766-5)
Supplement: Supplementary file 5 — Usual care table. (DOCX 19 kb) [file 13063_2018_2766_MOESM5_ESM.docx]

**Additional file 5: Usual care table to record adverse events and unplanned hospital admission/ the use of emergency care service** (please tick ✓ in the cells of adverse events you had)

Code of patient: ____________ Code of health facility: _____________

| **Items** | | **1^st^ month**  **____D__ __M____Y** | **2^nd^ month**  **____D__ __M____Y** | **3^rd^ month**  **____D__ __M____Y** | **4^th^ month**  **____D__ __M____Y** | **5^th^ month**  **____D__ __M____Y** | **6^th^ month**  **____D__ __M____Y** |
| --- | --- | --- | --- | --- | --- | --- | --- |
| **Adverse events** | falls and fall-related injuries |  |  |  |  |  |  |
|  | pins and needles |  |  |  |  |  |  |
|  | dizziness |  |  |  |  |  |  |
|  | headache |  |  |  |  |  |  |
|  | sleep difficulties |  |  |  |  |  |  |
|  | swelling of legs or ankles |  |  |  |  |  |  |
|  | chest distress |  |  |  |  |  |  |
|  | stomach disorder |  |  |  |  |  |  |
|  | backache |  |  |  |  |  |  |
|  | cough |  |  |  |  |  |  |
|  | dyspnoea |  |  |  |  |  |  |
|  | vomit |  |  |  |  |  |  |
|  | Others(please specify) |  |  |  |  |  |  |
| Numbers of unplanned hospital admission/ the use of emergency care service | |  |  |  |  |  |  |
